# Epigenetic changes driven by environmental pollutants in lung carcinogenesis: a comprehensive review

**DOI:** 10.3389/fpubh.2024.1420933

**Published:** 2024-10-08

**Authors:** Aijia Zhang, Xuexing Luo, Yu Li, Lunchun Yan, Xin Lai, Qianxu Yang, Ziming Zhao, Guanghui Huang, Zheng Li, Qibiao Wu, Jue Wang

**Affiliations:** ^1^Faculty of Humanities and Arts, Macau University of Science and Technology, Taipa, Macau SAR, China; ^2^State Key Laboratory of Quality Research in Chinese Medicines, Macau University of Science and Technology, Taipa, Macau SAR, China; ^3^Faculty of Chinese Medicine, Macau University of Science and Technology, Taipa, Macau SAR, China; ^4^Department of Comprehensive Surgery, Hengqin Hospital, The First Affiliated Hospital of Guangzhou Medical University, Guangdong-Macao in-Depth Cooperation Zone in Hengqin, Hengqin, China; ^5^Department of Traditional Chinese Medicine, The Sixth Affiliated Hospital, Sun Yat-sen University, Guangzhou, China; ^6^Centre for Epidemiology and Evidence-Based Practice, Department of Social and Preventive Medicine, Faculty of Medicine, University of Malaya, Kuala Lumpur, Malaysia; ^7^Jiangsu Engineering Research Center of Cardiovascular Drugs Targeting Endothelial Cells, College of Health Sciences, School of Life Sciences, Jiangsu Normal University, Xuzhou, Jiangsu Province, China; ^8^State Key Laboratory of Natural and Biomimetic Drugs, Peking University, Beijing, China; ^9^Guangdong-Hong Kong-Macao Joint Laboratory for Contaminants Exposure and Health, Guangzhou, Guangdong Province, China

**Keywords:** epigenetics, lung cancer, DNA methylation, environment pollution, miRNA

## Abstract

Lung cancer remains the leading cause of cancer-related mortality globally, with environmental pollutants identified as significant risk factors, especially for nonsmokers. The intersection of these pollutants with epigenetic mechanisms has emerged as a critical area of interest for understanding the etiology and progression of lung cancer. Epigenetic changes, including DNA methylation, histone modifications, and non-coding RNAs, can induce alterations in gene expression without affecting the DNA sequence and are influenced by environmental factors, contributing to the transformation of normal cells into malignant cells. This review assessed the literature on the influence of environmental pollutants on lung cancer epigenetics. A comprehensive search across databases such as PubMed, Web of Science, Cochrane Library, and Embase yielded 3,254 publications, with 22 high-quality papers included for in-depth analysis. These studies demonstrated the role of epigenetic markers, such as DNA methylation patterns of genes like F2RL3 and AHRR and alterations in the miRNA expression profiles, as potential biomarkers for lung cancer diagnosis and treatment. The review highlights the need to expand research beyond homogenous adult male groups typically found in high-risk occupational environments to broader population demographics. Such diversification can reduce biases and enhance the relevance of findings to various clinical contexts, fostering the development of personalized preventive and therapeutic measures. In conclusion, our findings underscore the potential of innovative epigenetic therapies, such as DNA demethylating drugs and histone modification agents, to counter environmental toxins’ carcinogenic effects. The growing interest in miRNA therapies and studies aiming to correct aberrant methylation patterns indicate significant strides toward better lung cancer management and a healthier future for global communities.

## 1 Introduction

Lung cancer is the most prevalent malignant tumor afflicting humanity, consistently occupying the highest rank in both global cancer incidence and mortality rates (1–3). According to GLOBOCAN estimates of incidence and mortality for 36 cancers in 185 countries worldwide, lung cancer is the most commonly diagnosed cancer in 2022, with nearly 2.5 million new cases and accounting for one in eight cancers worldwide (4). In our country, including the Macao Special Administrative Region, lung cancer ranks first in incidence and mortality rates among all malignant tumors (3, 5). Non-small cell lung cancer (NSCLC) comprises approximately 85% of all cases and includes adenocarcinoma, squamous cell carcinoma, and large cell carcinoma, among others (2, 6). Although smoking is a recognized primary risk factor for lung cancer, a significant number of lung cancer cases, particularly among Asian women who have never smoked, are associated with air pollution and environmental pollutants (7–9). These pollutants, including particulate matter, toxic metals, and nitrogen oxides, threaten everyone’s health (10–12). These pollutants penetrate the human respiratory system, potentially inducing epigenetic changes that lead to the transformation of normal cells into cancerous cells (13–16).

Due to the insidious onset of lung cancer, most patients are diagnosed at an advanced stage, missing the best opportunity for surgical treatment. Chemotherapy and targeted therapy are the main treatments for these patients. Chemotherapy often comes with many adverse reactions, and targeted therapy frequently leads to issues such as drug resistance, which results in many patients being unable to tolerate the drug treatment (10–12, 17). Over the past two decades, epigenetic research has advanced by leaps and bounds, offering a glimmer of hope for novel diagnostics and treatments of lung cancer. Epigenetics orchestrates the regulation of gene expression without altering the DNA sequence itself, thus revealing the intricate process of lung cancer formation from a genetic perspective. Alterations in epigenetics have been identified as crucial prognostic elements and potential therapeutic targets, with studies indicating that methylation patterns of specific genes, such as RASSF1A and RUNX3, are correlated with the prognosis and recurrence of lung cancer (16, 18). Epigenetic modifications, such as DNA methylation, histone modification, and non-coding RNAs, play a pivotal role in the onset and progression of lung cancer by regulating gene expression, impacting cell cycles, genomic imprinting, and X-chromosome inactivation (19–22). A deeper understanding of the underlying biological pathways elucidates how environmental pollutants induce such epigenetic changes. For instance, the p16INK4a pathway, often silenced by promoter hypermethylation, is crucial for cell cycle regulation and is frequently altered in lung carcinogenesis. Similarly, the PI3K/AKT signaling pathway can be activated by the demethylation of certain genes, contributing to tumorigenesis. Mediators such as reactive oxygen species (ROS) generated by pollutants can lead to oxidative stress, subsequently causing DNA damage and altered methylation patterns. Additionally, histone deacetylases (HDACs) and DNA methyltransferases (DNMTs) have been identified as critical enzymes that mediate these epigenetic changes, making them potential targets for therapeutic interventions. Hence, contemporary etiological studies of lung cancer are also focusing on the intersection of environmental pollutants and epigenetic mechanisms. An in-depth examination of these mechanisms provides novel strategies for treating lung cancer (23–25).

**Figure 1:**
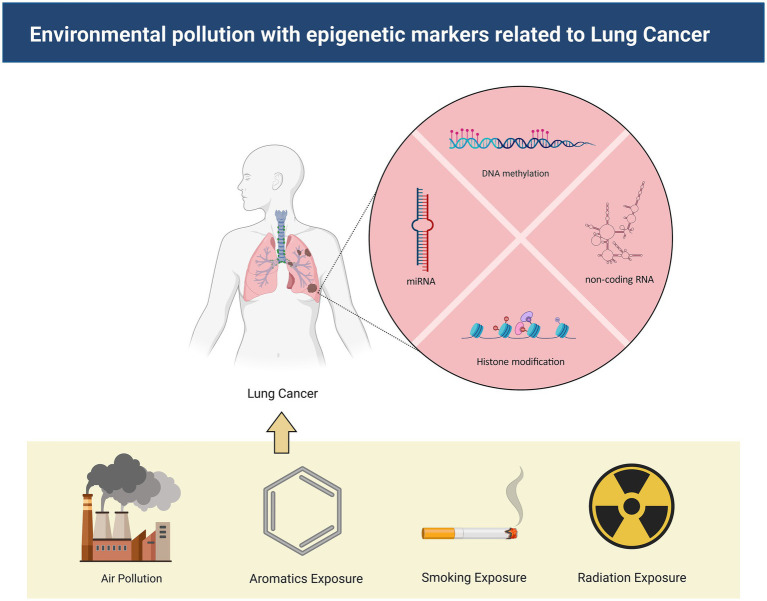
The mechanisms by which environmental pollutants lead to lung cancer. Environmental contaminants exert influence on non-coding RNA, DNA methylation, and histone modification, thereby instigating the onset of lung cancer.

As industrialization accelerates, the incidence and mortality rates of lung cancer have surged dramatically, necessitating the implementation of urgent public health measures and innovative research to prevent and combat this disease (26–28). This article encapsulates the impact of environmental pollutants on the epigenetic alterations associated with lung cancer, and the underlying physiological mechanisms induced by these contaminants (Figure 1). Understanding the molecular mechanics of epigenetic changes and their correlation with environmental pollutants can pave the way for the development of novel therapeutics and preventive measures for lung cancer, ultimately enhancing patient survival quality and prognosis, and prolonging patient lifespan (29–32).

## 2 Methods

### 2.1 Search strategy

**Figure 2:**
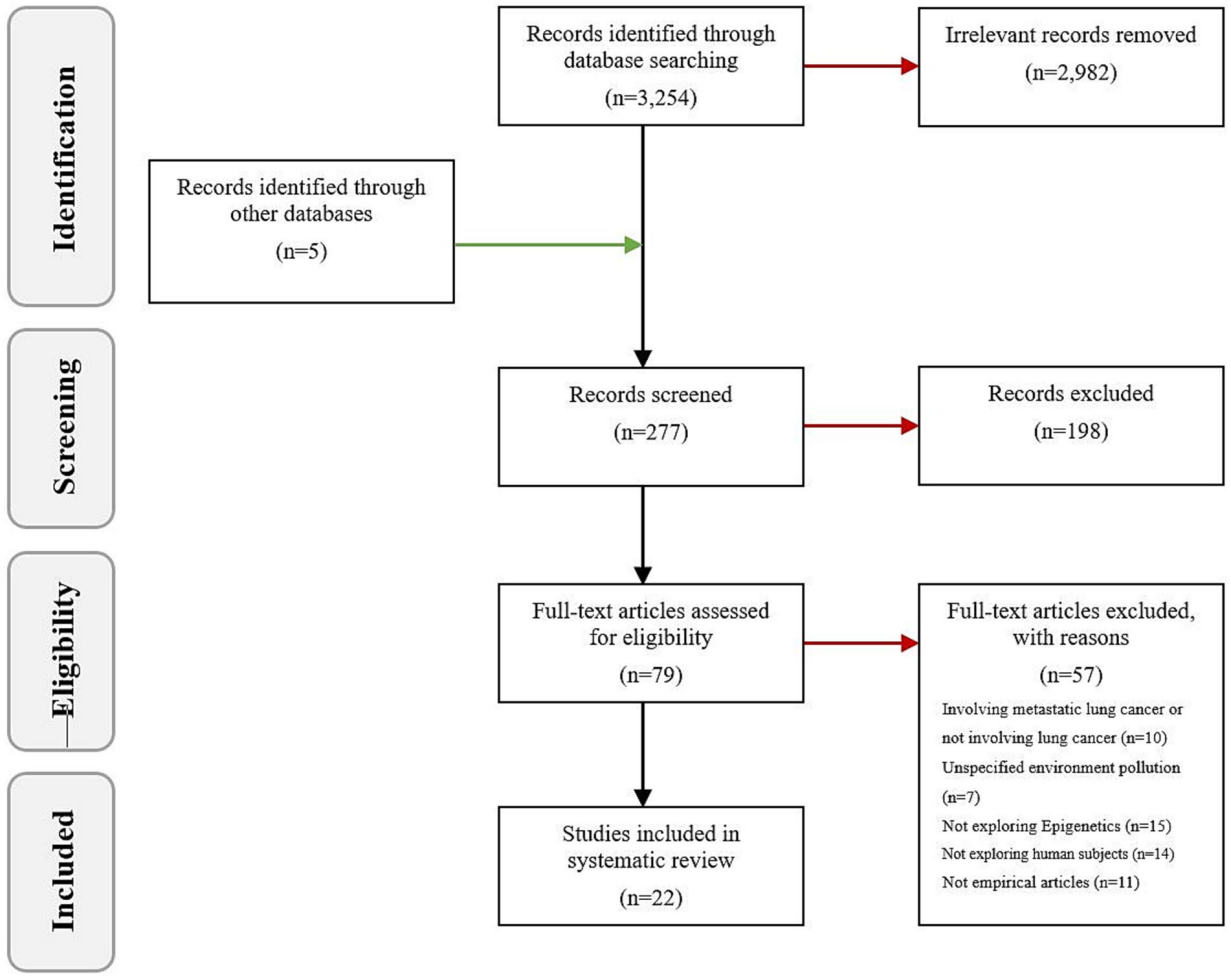
Flow diagram for the included and excluded articles.

Utilizing the electronic databases PubMed, Web of Science, Cochrane Library, and Embase, we carried out an exhaustive literature search for publications released prior to November 2023. The search was conducted using key terms that encompass the following: 1: environmental pollution (environmental biomarkers, air pollution, radiation, tobacco smoke pollution, aromatic hydrocarbons); 2: epigenetics (DNA methylation, histone, non-coding RNA); 3: lung cancer (primary bronchogenic carcinoma, non-small cell lung cancer, small cell lung cancer). The schema for the search is depicted in Table 1. Additionally, manual searches were also performed within the bibliographies of published articles and reviews. Adhering to the inclusion criteria, we discussed 22 high-quality papers from the initial pool of 3,254. The outcome of the search and the inclusion and exclusion process are shown in Figure 2.

**Table 1:** Search strategies for English databases or Chinese databases.

Number	Search terms
#1	Environmental Pollution [MeSH]
#2	Environmental Biomarkers [MeSH]
#3	Air Pollution [MeSH]
#4	Air Pollution, Radioactive [MeSH]
#5	Air Pollution, Indoor [MeSH]
#6	Tobacco Smoke Pollution [MeSH]
#7	Radiation [MeSH]
#8	Hydrocarbons, Aromatic [MeSH]
#9	#1 OR #2 OR #3 OR #4 OR #5 OR #6 OR #7 OR #8
#10	Epigenomics [MeSH]
#11	DNA Methylation [MeSH]
#12	#10 OR #11
#13	Lung Neoplasms [MeSH]
#14	Small Cell Lung Carcinoma (SCLC) [MeSH]
#15	Non small cell lung cancer (NSCLC) [MeSH]
#16	Lung Cancer Cell Lines[MeSH]
#17	#13 OR #14 OR #15 OR #16
#18	#9 AND #12 AND #17
#19	#9 keywords translated into Chinese
#20	#12 keywords translated into Chinese
#21	#17 keywords translated into Chinese
#22	#19 AND #20 AND #21

MeSH, Medical Subject Headings; The formular of Search by PubMed with: (“Environmental Pollution”[Mesh]) OR “(Environmental Biomarkers”[Mesh]) OR (“Air Pollution”[Mesh] OR “Air Pollution, Indoor”[Mesh] OR “Air Pollution, Radioactive”[Mesh]) OR “(Tobacco Smoke Pollution”[Mesh]) OR “(Radiation”[Mesh]) OR “(Hydrocarbons, Aromatic”[Mesh]) AND “(Epigenomics”[Mesh]) OR “(DNA Methylation”[Mesh]) AND “(Lung Neoplasms”[Mesh]) OR “(Small Cell Lung Carcinoma”[Mesh]) OR “(Carcinoma, Non-Small-Cell Lung”[Mesh]) OR “Lung Cancer Cell Lines”[Mesh].

### 2.2 Inclusion and exclusion criteria

The criteria for inclusion of literature were as follows: (1) Studies that exclusively investigate primary bronchogenic carcinoma; (2) Research defining air pollution factors according to the “Environmental Health” (33) criteria established by the World Health Organization, which includes environmental biomarkers, air pollution, radiation, tobacco smoke pollution, and aromatic hydrocarbons; (3) Studies involving any epigenetic mechanisms, inclusive of DNA methylation, histone modifications, and non-coding RNA; (4) Research conducted in human subjects; (5) The study designs included analyses of cohort, cross-sectional and longitudinal studies, as well as randomized, non-randomized and semi-randomized studies.

Exclusion criteria were: (1) Literature pertaining to metastatic lung cancer or studies not concerning lung cancer; (2) Studies without relevant environmental pollutant exposure; (3) Research lacking examination of epigenetic mechanisms; (4) Animal studies; (5) Academic theses, conference abstracts, books, reports, or non-empirical articles.

### 2.3 Data extraction

Two reviewers, A.J. Zhang and X.X. Luo, independently screened the titles, abstracts, and full texts of the retrieved articles, and sorted out the studies that met the inclusion criteria. Any disagreements between reviewers were resolved by discussion and reaching a consensus, with the contribution of a third independent reviewer, Y. Li, made the final decision when necessary.

Data were extracted from the included literature using a standardized data extraction form. The collected information included: (1) Basic details: author’s name, year of publication, region of publication, and number of cases included; (2) Type of study; (3) Clinical and pathological data of participants; (4) Experimental methods; (5) Outcome measures.

### 2.4 Assessment of evidence quality

As the included articles employed disparate methodological approaches, we employed a multi-method quality framework to assess the quality of the articles according to standardized criteria (34). The framework was categorized into four main quality categories: truth value, applicability, consistency and neutrality. In addition, we considered the context of the study, potential benefits and harms, and patient value systems when interpreting the results. A score was assigned to each category, with the average score across the four categories indicating that the overall quality of the article was rated as robust, high, moderate, low, or very low.

## 3 Results

The search terms identified a total of 3,254 articles. A rigorous selection process led to the exclusion of some studies: 10 concerning metastatic lung cancer or not involving lung cancer, 7 with undefined types of environmental pollutants, 15 lacking examinations of epigenetic mechanisms, 14 based on non-human subjects, and 11 non-empirical articles. Ultimately, 22 published studies met the predetermined inclusion criteria (Table 2).

**Table 2:** Record of citation analyses and full texts reviewed.

Name	Region/Country	Types	Participants	Exposure	Methodology	Analysis	Results
Alhamdow et al. (35)	Sweden	Research article	151 chimney sweeps, 19 creosote-exposed workers and 152 unexposed workers (controls), all men	PAHs	Measured monohydroxylated metabolites of phenanthrene and fluorene in urine using liquid chromatography-mass spectrometry.	Unadjusted and multivariable linear regression models were fit to evaluate associations.	Increasing fluorene exposure, among chimney sweeps, was associated with lower DNA methylation of *F2RL3* and *AHRR*, markers for increased lung cancer risk.
Baglietto, et al. (36)	France	Research article	Participants were from the EPIC-Italy cohort and the MCCS cohort, including cases of lung cancer and controls, with over 700 case–control pairs in total.	Tobacco	Used Illumina Infinium HumanMethylation450 array to measure DNA methylation in pre-diagnostic blood samples.	Conditional logistic regression models, stratified by smoking status, and fixed effect models for pooled ORs.	Identified six CpGs associated with lung cancer risk, hypomethylation observed in current smokers, and increased methylation post-quitting.
Fasanelli et al. (37)	Italy	Research article	132 case–control pairs in the NOWAC cohort and an additional 664 case–control pairs tightly matched for smoking from the MCCS, NSHDS and EPIC HD cohorts.	Tobacco	Genome-wide DNA methylation analyses were performed on pre-diagnostic blood samples using the Illumina Infinium HumanMethylation450 platform.	Performed mediation analysis to assess whether methylation of cg05575921 (AHRR) and cg03636183 (F2RL3).	The most significant associations with lung cancer risk are for cg05575921 in *AHRR* and cg03636183 in *F2RL3*, previously shown to be strongly hypomethylated in smokers. These associations remain significant after adjustment for smoking.
Guo et al. (40)	China	Review article	Truck drivers and office workers in Beijing	PMs	Multilevel mixed-effect regression models	The data were analyzed using multilevel mixed-effect regression models to account for the lack of independence between repeated measures.	Interquartile increases in personal PM2.5 and ambient PM10 levels were associated with significant covariate-adjusted decreases in SATa methylation.
Guo et al. (53)	China	Review article	N/A	PM2.5	Epidemiological and toxicological studies, biomarker investigations.	N/A	Results indicate PM2.5 exposure is associated with oxidative stress, inflammation, DNA damage, and epigenetic changes, potentially leading to respiratory diseases.
Hammons et al. (51)	USA	Research article	55 human donors (smokers and nonsmokers)	Tobacco	RT-PCR analysis, DNA MTase enzyme assay	Data were analyzed statistically by ANOVA using Sigma-Stat software, with Tukey test evaluating differences between means.	DNA MTase mRNA levels were significantly higher in smokers.
Hou et al. (41)	China	Research article	60 truck drivers and 60 office workers in Beijing	PM	Blood DNA methylation measured, personal exposure assessment	GEE models adjusted for covariates, FDR applied	Positive associations between PM elemental components and DNA methylation changes in a Beijing population, with NBL2 methylation linked to silicon (Si) and calcium (Ca) in truck drivers, and SATa methylation linked to sulfur (S) in office workers.
Huang et al. (39)	China	Research article	87 lung cancer patients and 31 healthy subjects	Smoky coals	Genomic DNA extracted from tissues and plasma; candidate gene promoter methylation status determined using Nested Methylation-Specific PCR (nMSP).	Sanger sequencing verified nMSP results; methylation frequencies compared across tissue and plasma samples.	Seven of eight genes showed high methylation frequencies in tissues (39–74%). Methylation in plasma was detected for five genes with frequencies of 45% for CDKN2A, 48% for DLEC1, 76% for CDH1, 14% for DAPK, and 29% for RUNX3. Healthy controls showed no methylation.
Jabeen, M et al. (42)	USA	Research article	A549 lung carcinoma cells	10-, 200-, and 400 μM concentrations of PFAS	Cell culture, MTT assay, qRT-PCR, UPLC-MS, HS-DFM.	Used GraphPad Software for statistical tests and analysis.	Exposure to per- and polyfluoroalkyl substances (PFAS) can cause epigenetic modifications in A549 lung cancer cells. Lower doses of PFAS compounds promote cell proliferation, whereas higher concentrations induce apoptosis, potentially impacting patients with pre-existing lung conditions or contributing to lung carcinogenesis.
Lee et al. (38)	South Korea	Research article	330 adults (46 to 87 years of age)	Tobacco	Pyrosequencing was performed to measure DNA methylation of AHRR and F2RL3.	The Kruskal-Wallis ANOVA test was used to compare data. Pearson tests were performed to assess any correlation between methylation values.	AHRR and F2RL3 genes were significantly hypomethylated in current smokers. AHRR methylation is significantly associated with the risk of lung cancer (OR = 0.96, *p* = 0.011).
Li et al. (54)	China	Review article	N/A	PMs	English-language publications focusing on PM, epigenetic changes, and lung cancer were reviewed.	Reviewing English-language publications and conducting a comprehensive comparison approach.	PM2.5 is associated with the increased lung cancer risk and mortality. PM-induced epigenetic changes may play important roles in the pathogenesis of lung cancer.
Liang et al. (43)	China	Research article	19–23 years old students	PM2.5	Mixed-effects models were used to evaluate the influence of PM2.5 and its constituent exposure on DNAm while controlling for potential confounders.	Used MethylTarget to determine and analyze DNAm of imprinted genes in blood samples. Statistical analysis included natural logarithmic transformation of methylation data and mixed-effects models.	No significant correlation between DNAm and personal PM2.5 exposure mass. However, DNAm changes in eight imprinted control regions (ICRs) and a non-imprinted gene were significantly associated with PM2.5 constituents.
Mukherjee et al. (55)	India	Review article	N/A	Air pollution	Literature review and analysis of 235 articles	N/A	DNA methylation represents the most prominent epigenetic alteration underlying the air pollution-induced pathogenic mechanism. Several other types of epigenetic changes, such as histone modifications, miRNA, and non-coding RNA expression, have also been found to have been linked with air pollution.
Pan et al. (45)	China	Research article	105 patients with untreated lung adenocarcinoma (AD) or squamous cell carcinoma (SCC)	Smoky coals	MicroRNA microarray analysis, quantitative RT-PCR, cell culture assays, luciferase reporter assays, animal studies	Volcano Plot filtering, Median normalization, Student’s t-test, Pearson correlation analysis	miR-144 was significantly down-regulated in NSCLCs from HPR; miR-144 targets oncogene Zeb1; overexpression of miR-144 inhibits NSCLC cell migration and tumor progression.
Sanchez-Guerra et al. (52)	USA	Review article	60 truck drivers, 60 office workers in Beijing	PMs	ELISA for global 5mC and 5hmC; mixed-effects regression models	Adjusted mixed-effects regression models were used to evaluate associations.	PM10 exposure associated with increased 5hmC levels, no correlation with 5mC.
Sato & Ishigami (44)	Japan	Review article	Human lung adenocarcinoma (A549) cells	HTPs, RC	Cell treatment with aerosol extracts, global DNA methylation analysis, gene expression profiling.	Cell culture treated with aerosol extracts, followed by various assays (dot blot, RRBS, DNA microarray, RT-qPCR).	The HTP extract affected gene expression. In particular, the HTP extract markedly affected the mRNA expression and promoter methylation of cytochrome P450 family 1 subfamily A member 1 (CYP1A1), which is associated with carcinogenic risk.
Schembri et al. (46)	USA	Research article	20 volunteers (10 current smokers, 10 never smokers)	Tobacco	The methodological approach of the study involved microarray profiling of miRNAs and mRNAs, *in vitro* transfections to modulate miRNA levels, and real-time PCR validations to assess the effects on gene expression changes in response to cigarette smoke exposure.	The article analyzed data using microarray preprocessing, normalization, Welch’s t-test, and Pearson correlation, followed by GSEA and hierarchical clustering.	The study found 28 miRNAs differentially expressed in smokers, with mir-218 significantly down-regulated, which modulates airway epithelial gene expression response to cigarette smoke.
Sima et al. (47)	Czech Republic	Review article	N/A	Air pollution	Literature review, data synthesis, and analysis of miRNA deregulation in relation to air pollution and lung cancer.	Data analysis involved literature search, miRNA pattern comparison, and identification of commonalities in miRNA deregulation.	Detected a total of25 miRNAs meeting the criteria, among them, miR-222, miR-21, miR-126-3p, miR-155 and miR-425 being the most prominent.
Tellez et al. (48)	USA	Research article	Immortalized human bronchial epithelial cells (HBEC)	Tobacco	*In vitro* model, gene expression analysis, immunoblot, chromatin immunoprecipitation assay.	qRT-PCR, immunoblot, chromatin immunoprecipitation, bisulfite sequencing, statistical analysis using Pearson correlation and t-tests.	Carcinogen exposure induces EMT and stem cell-like properties in HBECs through epigenetic silencing of miR-200 and miR-205.
Wang et al. (49)	USA	Research article	Healthy nonsmokers and healthy smokers	Tobacco	miRNA microarray analysis, qRT-PCR validation, bioinformatics tools	Processed Affymetrix miRNA array data using Partek, performed two-way ANOVA, and validated with qRT-PCR.	The study found that smoking induces persistent dysregulation of 12 miRNAs in the small airway epithelium even after smoking cessation, which may contribute to the increased risk of COPD and lung cancer in former smokers.
Wu et al. (56)	China	Systematic review and meta-analysis	38 articles were included in this study: 16 using global methylation, 18 using candidate genes, and 11 using EWAS, with 7 studies using more than one approach.	Air pollution	Systematic search, meta-analysis, and candidate-gene, epigenome-wide association studies (EWAS)	Meta-analysis, heterogeneity assessed with Cochran Q test and I^2^ statistic, sensitivity and publication bias tests using R Studio and Stata.	Imprecise inverse association between PM2.5 and global DNA methylation; candidate-gene results suggest hypermethylation in ERCC3 with benzene and SOX2 with PM2.5 exposure; 201 CpG sites and 148 differentially methylated regions associated with air pollution.
Xi et al. (50)	USA	Research article	Normal human respiratory epithelial cells and lung cancer cells	Tobacco	Array techniques, qRT-PCR, Ago-CLIP, luciferase assays, ChIP, MeDIP, MNase protection	Methodology for data analysis includes qRT-PCR, Western blot, Ago-CLIP, luciferase reporter assays, ChIP, MeDIP, MNase protection assays, and statistical tests.	These findings indicate that miR-487b is a tumor suppressor microRNA silenced by epigenetic mechanisms during tobacco-induced pulmonary carcinogenesis and suggest that DNA demethylating agents may be useful for activating miR-487b for lung cancer therapy.

PAHs, polycyclic aromatic hydrocarbons; PMs, particulate matter; HTPs, Aerosol extracts of heated tobacco products; RC, reference cigarette.

### 3.1 Characteristics of the included studies

Tables 2, 3 summarize the detailed characteristics of the included studies. According to quality assessment standards, six studies were of good quality, and 16 were of moderate quality. The identified research employed a variety of methods to detect alterations in lung cancer epigenetics: 16 studies conducted assays of specific candidate genes as shown in Table 4, which included *F2RL3* and *AHRR* (35–38), CDKN2A, DLEC1, CDH1, DAPK, RUNX3, APC, WIF1, and MGMT (39), SATα, NBL2, and D4Z4 (40, 41), DNMT1, DNMT3a, DNMT3b, TET1, TET2, TET3 (42), L3MBTL1, NNAT, PEG10, GNAS Ex1A, MCTS2, SNURF/SNRPN, IGF2R, RB1, and CYP1B1 (43), CYP1A1 (44), miRNA (45–50). Additionally, some studies assessed the impact of environmental pollutants using different mediators, such as raised levels of DNA methyltransferase enzyme (DNA MTase) (51) and chemical modifications 5mC and 5hmC (52); 1 study conducted a whole-genome DNA methylation analysis using the Illumina Infinium HumanMethylation450 platform (36); 4 studies reviewed the effect of environmental pollutants on epigenetics (53–56).

**Table 3:** Record of citation score.

	Validate	Suitability	Therapeutic	Consistency	Overall score
Alhamdow et al. (35)	5	5	4	5	Good (5)
Baglietto, et al. (36)	4	4	3	4	Moderate (4)
Fasanelli et al. (37)	4	5	3	4	Moderate (4)
Guo et al. (40)	3	3	2	4	Moderate (3)
Guo et al. (53)	5	5	4	4	Good (5)
Hammons et al. (51)	5	5	4	5	Good (5)
Hou et al. (41)	3	3	2	4	Moderate (3)
Huang et al. (39)	5	4	3	4	Moderate (4)
Jabeen, M et al. (42)	4	5	3	4	Moderate (4)
Lee et al. (38)	5	5	4	5	Good (5)
Li et al. (54)	4	5	3	4	Moderate (4)
Liang et al. (43)	4	4	3	4	Moderate (4)
Mukherjee et al. (55)	5	5	4	5	Good (5)
Pan et al. (45)	4	4	4	5	Moderate (4)
Sanchez-Guerra et al. (52)	3	2	3	3	Moderate (3)
Sato & Ishigami (44)	4	4	3	4	Moderate (4)
Schembri et al. (46)	4	3	3	4	Moderate (4)
Sima et al. (47)	4	4	2	3	Moderate (3)
Tellez et al. (48)	4	3	3	3	Moderate (3)
Wang et al. (86)	5	5	4	5	Good (5)
Wu et al. (56)	4	3	3	3	Moderate (3)
Xi et al. (50)	4	4	5	4	Moderate (4)

**Table 4:** The types of candidate genes for detecting alterations in lung cancer epigenetics.

Candidate gene type	Authors	Exposure	General characteristics of epigenetic changes
F2RL3, AHRR	Alhamdow et al. (35)	PAHs	DNA methylation
	Baglietto, et al. (36)	Tobacco	
	Fasanelli et al. (37)	Tobacco	
	Lee et al. (38)	Tobacco	
CDKN 2A, DLEC 1, CDH 1, DAPK, RUNX 3, APC, WIF 1 and MGMT	Huang et al. (39)	Smoky coals	
SATα, NBL2 and D4Z4	Guo et al. (40)	PMs	
	Hou et al. (41)	PM	
DNMT1, DNMT3a, DNMT3b, TET1, TET2, TET3	Jabeen et al. (42)	10-, 200-, and 400 μM concentrations of PFAS	
L3MBTL1, NNAT, PEG10, GNAS, Ex1A, MCTS2, SNURF/ SNRPN, IGF2R, RB1 and CYP1B1	Liang et al. (43)	PM2.5	
CYP1A1	Sato & Ishigami, (44)	HTPs, RC	
HATs and HDACs	Guo et al. (53)	PM2.5	Histone modifications
Kdm6a	(55)	Air pollution	
miRNA	Guo et al. (53)	PM2.5	microRNAs
miRNA	Li et al. (54)	PM	
miR-144	Pan et al. (45)	Smoky coals	
mir-218	Schembri et al. (46)	Tobacco	
miR-222, miR-21, miR-126-3p, miR-155, and miR-425	Sima et al. (47)	Air pollution	
miR-196b, miR-200, and miR-205	Tellez et al. (48)	Tobacco	
miRNA	Wang et al. (49)	Tobacco	
miR-487b	Xi et al. (50)	Tobacco	

PAHs, polycyclic aromatic hydrocarbons; PMs, particulate matter; HTPs, Aerosol extracts of heated tobacco products; RC, reference cigarette.

### 3.2 Participant demographics

The inquiries predominantly explored the demographic of male adults, with a preponderance of professions including drivers and laborers. These investigations unveiled a considerable overlap in samples, as three studies (40, 41, 52) recruited truck drivers from China for analysis. The pathological phenotypes were most frequently assessed through DNA methylation patterns, some of which involved levels of gene expression. However, other epigenetic pathways, such as histone modifications or non-coding RNAs, have yet to be thoroughly examined.

### 3.3 Types of pertinent environmental pollutants

According to the research conducted by Xue et al. (57), environmental pollutants associated with lung cancer are categorized into two types: outdoor and indoor air pollutants (Table 5).

**Table 5:** Types of environmental pollutants that trigger epigenetic changes in lung cancer.

Type	Authors	Environmental pollutants	Epigenetic changes in lung cancer
Outdoor Air Pollutants	Alhamdow et al. (35)	PAHs	PAHs induced hypomethylation of F2RL3 and AHRR, epigenetic changes linked to lung cancer risk.
	Hou et al. (41)	PM	PM exposure induced hypomethylation in tandem repeats SATa and NBL2 among study participants, potentially impacting lung cancer risk.
	Guo et al. (40)	PMs	PM exposure is linked to hypomethylation of tandem repeats SATa, NBL2, and D4Z4, potentially impacting lung cancer risk.
	Guo et al. (53)	PM2.5	PMs induces epigenetic alterations such as DNA methylation, histone modification, and miRNA dysregulation, contributing to lung carcinogenesis.
	Li et al. (54)	PM	
	Sanchez-Guerra et al. (52)	PMs	PM10 exposure linked to increased blood 5-hydroxymethylcytosine (5hmC), indicative of epigenetic changes in lung cancer risk.
	Liang et al. (43)	PM2.5	PM2.5 exposure induced changes in DNA methylation of imprinted genes, potentially affecting lung cancer pathways and susceptibility.
Indoor Air Pollutants	Baglietto, et al. (36)	Tobacco	Smoking exposure induced hypomethylation of AHRR and F2RL3, associated with increased lung cancer risk.
	Fasanelli et al. (37)	Tobacco	
	Lee et al. (38)	Tobacco	
	Hammons et al. (51)	Tobacco	Tobacco was associated with increased expression of hepatic DNA methyltransferase, which indicate a greater susceptibility to cancer.
	Schembri et al. (46)	Tobacco	Tobacco induce down-regulation of miR-144, affecting Zeb1 expression and promoting epithelial-mesenchymal transition in lung cancer cells.
	Sato & Ishigami (44)	HTPs, RC	RC reduced 5-mC and 5-hmC; HTPs altered CpG, affecting CYP1A1 mRNA and methylation, linked to cancer risk.
	Tellez et al. (48)	Tobacco	Tobacco induces epigenetic changes including promoter hypermethylation and H3K27me3 enrichment, leading to silencing of tumor-suppressive miRNAs.
	Wang et al. (86)	Tobacco	Tobacco induces epigenetic repression of miR-487b and alters microRNA expression, contributing to lung carcinogenesis.
	Xi et al. (50)	Tobacco	Tobacco induces demethylation of miR-487b, alters nucleosome positioning, and increases DNA methylation, leading to its repression and lung cancer progression.
	Huang et al. (39)	Smoky coals	Smoky coals induced aberrant methylation in promoters of lung cancer-related genes, potentially serving as epigenetic biomarkers for early detection.
	Pan et al. (45)	Smoky coals	Smoky coals induced down-regulation of miR-144, associated with increased Zeb1 expression and EMT phenotype in lung cancer.

HTPs, Aerosol extracts of heated tobacco products; RC, reference cigarette.

#### 3.3.1 Outdoor air pollutants

Eight studies identified within the pertinent body of research analyzed the hazards posed by outdoor environmental pollutants, focusing primarily on particulate matter (PMs) and polycyclic aromatic hydrocarbons (PAHs) (35, 40, 41, 43, 45, 52, 53, 58). The composition of atmospheric particulates is complex, encompassing organic compounds (such as polycyclic aromatic hydrocarbons, dioxins, and benzene) and inorganic substances (like carbon, chlorides, nitrates, and sulfates) and metals. Due to their substantial surface area and robust adsorption capacity, PMs not only carry toxic metals and organic constituents but can also adsorb bacteria and virus (59). These pollutants enter the lungs via the respiratory system and could potentially elevate the risk of developing lung cancer.

#### 3.3.2 Indoor air pollutants

An additional 10 studies discussed the possibility of indoor environmental pollutants—including tobacco smoke and coal for curing—inducing epigenetic modifications associated with lung cancer (36–39, 44, 46, 48–51). Indoor smoking and exposure to secondhand smoke are significant risk factors for lung cancer. Long-term exposure to environmental tobacco smoke, including secondhand aerosols from tobacco or electronic cigarettes, increases the risk of lung cancer (49, 51). Moreover, some reports suggest that the cumulative toxicity of co-existing air pollutants is an important consideration to take into account (57).

### 3.4 Alterations in the epigenetics of lung cancer

**Figure 3:**
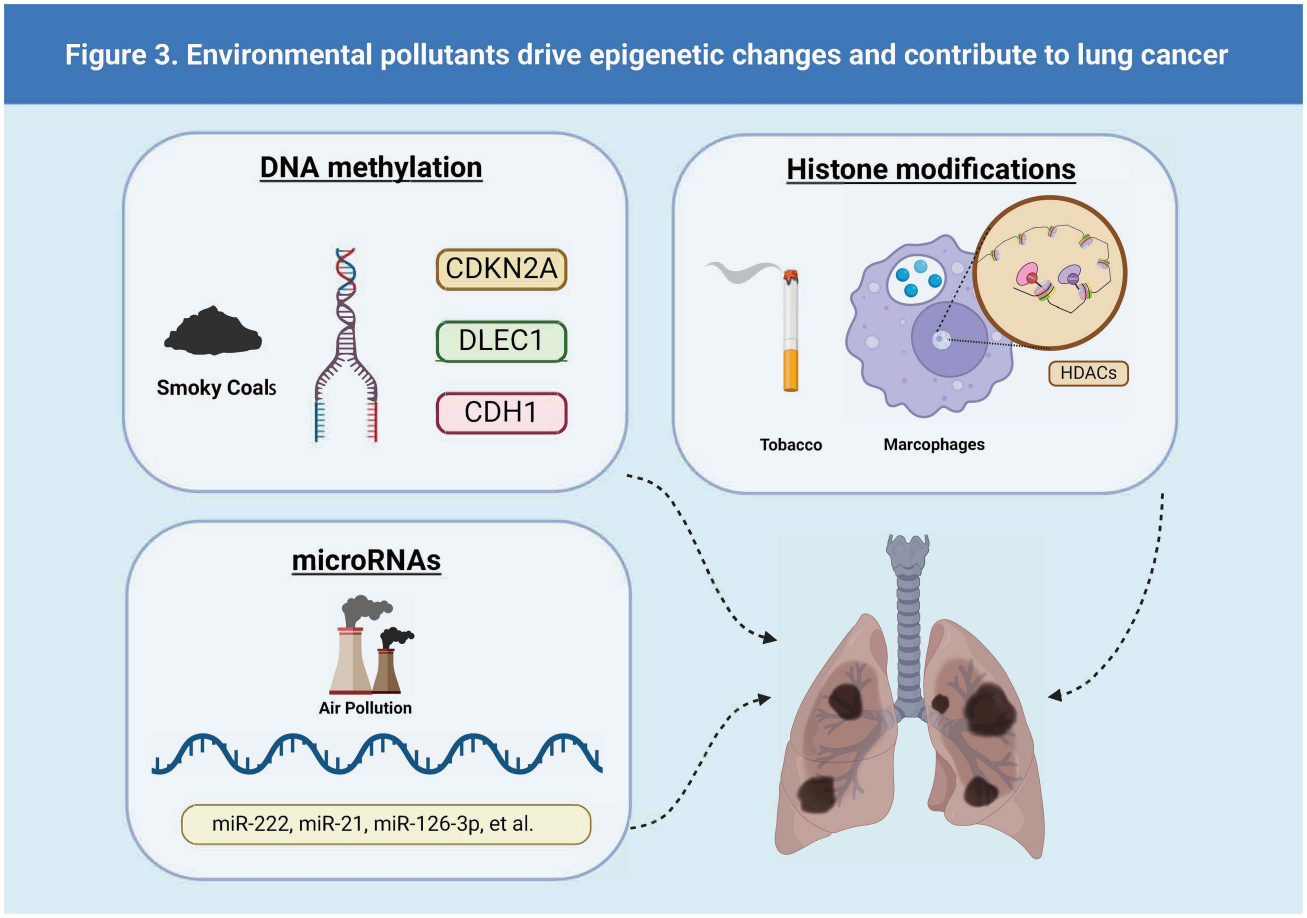
Environmental pollutants drive epigenetic changes and contribute to lung cancer.

Epigenetic alterations—including changes in microRNAs (miRNAs), DNA methylation, and histone modifications—are major determinants in the development of disease phenotypes following exposure to air pollution (53, 55). The different types of lung cancer driven by epigenetic changes driven by environmental pollutants are shown in Figure 3.

#### 3.4.1 The mechanisms of DNA methylation in driving lung cancer

DNA methylation represents a significant epigenetic change underlying the pathogenic mechanisms induced by air pollution (55). Changes in DNA methylation occurred after exposure to PMs (56). PM2.5 exposure suppresses p53 expression through promoter hypermethylation mediated by the ROS-protein kinase B (Akt)-DNMT3B pathway, suggesting that PM2.5 exposure could increase the risk of lung cancer (53). Furthermore, compounds produced by smoking (36–38) and perfluoroalkyl substances [PFAS, (42)] have been shown to affect gene expression in lung cancer cells by altering DNA methylation patterns. This alteration may lead to dysregulation of the cell cycle and apoptosis pathways, thereby promoting the onset and progression of lung cancer. The A549 lung cancer cell line serves as a research model, providing crucial experimental evidence for understanding how these environmental factors impact lung cancer (42, 44).

#### 3.4.2 The mechanisms of histone modifications in driving lung cancer

Some studies also show that differential histone modifications involve PM-induced inflammatory responses and oxidative stress, particularly leading to pulmonary diseases (53). Long-term exposure to PM2.5 downregulates the expression of histone demethylase Kdm6a in lung macrophages, which may result in increased methylation of H3K4 and H3K9 in the promoter regions of IL-6 and IFN-*β*. Exposure to cigarette smoke reduces the activity of histone deacetylases (HDACs) and decreases the expression of HDAC1, HDAC2, and HDAC3 in macrophages, resulting in an inflammatory response. Exposure to particulates leads to an imbalance in the expression of histone acetyltransferases (HATs) and HDACs in human bronchial epithelial cells, as well as an increase in the acetylation of certain histones, such as H4, which in turn triggers inflammation (55).

#### 3.4.3 The mechanisms of miRNAs in driving lung cancer

Sima et al. (47) analyzed the expression of miRNAs associated with exposure to air pollutants and lung cancer. Twenty-five miRNAs were correlated with exposure to air pollution and lung cancer, with miR-222, miR-21, miR-126-3p, miR-155, and miR-425 being the most significant. They play pivotal roles in promoting or inhibiting angiogenesis, inflammation, and the progression of lung cancer. Additionally, a specific set of upregulated or downregulated miRNAs was observed in the progression of bronchogenic carcinoma in smokers, ranging from normal lung to hyperplasia, metaplasia, carcinoma *in situ*, and finally, to lung squamous cell carcinoma (LUSC) (46). Exposure to cigarette smoke leads to an early, pronounced reduction in the expression of the tumor-suppressor miR-487b through promoter methylation, thereby facilitating lung oncogenesis through Wnt signaling (50). Similarly, tumor suppressor miR-196b is silenced early through promoter methylation in the same experimental model, giving a selective growth advantage to precancerous cells. A case–control study showed a strong correlation between methylation of miR-196b in sputum and the occurrence of lung cancer (48).

## 4 Discussion

Epigenetic characteristics mirror the shifts in cellular environments and are also discernible within the human circulatory system across a spectrum of diseases (60, 61). Exploration of these epigenetic attributes may lead to the identification of sensitive biomarkers, which hold promise for the early screening of lung cancer as well as the monitoring of the clinical treatment outcomes. This study is dedicated to revisiting the extensive interplay between environmental pollutants and lung cancer, with a comprehensive analysis of current research highlighting the crucial role that epigenetic modifications play in the etiology of lung cancer. Specifically, DNA methylation of genes such as F2RL3 and AHRR is accentuated (62–65), aberrant miRNA expression patterns stand out as additional key epigenetic markers (49, 66–68). In the future, these could serve as potential targets for diagnosing and treating lung cancer. However, such studies are predominantly confined to specific demographics, primarily consisting of adult males exposed to highly polluted environments, which may introduce biases and affect the objectivity of the data.

Corresponding research indicates that the impact of environmental pollutants on diverse populations is multifaceted, with contributing factors encompassing genetic characteristics, occupation, lifestyle choices, and socioeconomic status (69). In particular, alterations in DNA methylation at specific genomic loci constitute a fundamental aspect of the initiation and progression of lung cancer (70–73). Epigenetic variations observed within the CDKN2A gene, engendered by exogenous environmental elements, exemplify the paradigmatic mechanisms of tumor formation instigated by external environmental factors through the genesis of heterotypic cells (74).

The horizon of avant-garde therapeutic approaches brims with potential. Although minimization of exposure remains an unwavering pillar, the advent of molecular treatment regimens, ingeniously devised to rectify epigenetic aberrations, heralds a significant leap forward in therapeutic innovation. Pertaining to DNA demethylation (75–77), histone modification pharmacologic (75, 78, 79) and miRNA therapeutic interventions (54, 80, 81). Research into histone-modifying drugs and miRNA therapies may revolutionize the treatment approaches for individuals exposed to environmental toxins, heralding a paradigm shift in managing pollution-related lung cancer. The development of these treatments necessitates rigorous investigations to ascertain their safety and efficacy. Clinical trials examining the effectiveness of agents like azacitidine in correcting methylation patterns associated with pollution-induced lung malignancies are imperative (82, 83), as well as clinical trials evaluating the effectiveness of drugs like azacitidine in correcting methylation patterns in pollution-related lung cancers. Further assessment of the anti-inflammatory properties of HDAC inhibitors is also imperative (84, 85). Such endeavors in therapeutic experimentation bear the potential to catalyze transformative changes in care for individuals plagued by environmental toxins. Consequently, the research must be conducted meticulously to ensure beneficial outcomes.

### 4.1 Limitations

The limitations of our review merit recognition and warrant attention. Initially, the caliber of evidence extracted from the 18 documents included was heterogeneous, with some studies potentially needing more rigorous methodological design, comprehensive data collection, or extensive peer-review processes. Such imperfections in quality may impinge upon the reliability and universality of the research findings, as lower quality investigations could introduce biases or overlook critical variables, our literature search was confined solely to published articles in English, introducing a language bias that may have excluded pertinent studies published in other tongues, which could provide insights into the epigenetic impacts of environmental pollutants on lung cancer. Consequently, our findings do not encompass the complete scope of global research and may lead to an incomplete understanding of the subject matter.

The robustness of the discussions presented might also be questioned, as they may not have considered all alternative explanations, counterarguments, or the full breadth of complex interactions between environmental pollutants and genetic susceptibility across different populations. The discussions may also need more comprehensiveness in resolving the heterogeneity of the study populations and methodologies, potentially limiting the strength of the conclusions drawn.

These limitations underscore the necessity for a cautious interpretation of the review outcomes. Future research should strive to include a broader scope of studies, encompassing multiple languages and more diverse populations, to offer a more comprehensive understanding of the effects of environmental pollutants on lung cancer through epigenetic alterations. Furthermore, ensuring that discussions in future reviews are grounded in extensive consideration of all pertinent factors and opposing viewpoints will enhance the research findings’ validity and practical applicability.

## 5 Conclusion

The current review delves into an increasing body of evidence that underscores how environmental pollutants act as catalysts for carcinogenesis within pulmonary tissues, focusing on epigenetic mechanisms. Studies on epigenetic markers—particularly DNA methylation of pivotal genes such as F2RL3 and AHRR, as well as alterations in miRNA profiles affecting gene expression—have emerged as significant indicators for diagnosing and treating lung cancer. However, focusing solely on homogenous male adult populations within specific high-risk occupational environments may fall short of a comprehensive picture, as it fails to encapsulate the demographic and occupational diversity prevalent in a broader population base. Additionally, as suggested by prior comprehensive reviews, these epigenetic characteristics may extend beyond the biomarkers for lung cancer, representing the organism’s response to environmental stressors.

In light of these findings, it is imperative to expand the research scope to include more diverse population groups, thereby mitigating the risk of biased data that may not represent the entirety of vulnerable cohorts. Widening the demographic reach of these studies can greatly enhance the validity of research outcomes and facilitate their application across varied clinical settings. Moreover, it allows for formulating of personalized preventive measures and interventions, considering the intricate interplay products between unique epigenomic landscapes, environmental exposures, lifestyles, and genetic susceptibilities.

Looking ahead, the pursuit of innovative treatments such as drugs targeting DNA demethylation and histone modification offers new avenues for combatting pollution-induced malignancies. Rigorous scrutiny and clinical trials of these emerging therapeutic modalities, coupled with the burgeoning interest in miRNA therapies, highlight their potential to significantly impact on individuals affected by the deleterious effects of environmental toxins. Research aimed at correcting aberrant methylation patterns with drugs like azacitidine, as well as exploring the anti-inflammatory properties of HDAC inhibitors represent scientific endeavors and steps toward a healthier future for the global community.
